# Single‐cell analysis revealed a potential role of T‐cell exhaustion in colorectal cancer with liver metastasis

**DOI:** 10.1111/jcmm.18341

**Published:** 2024-04-22

**Authors:** Tianlong Ling, Cheng Zhang, Ye Liu, Chunhui Jiang, Lei Gu

**Affiliations:** ^1^ Department of Gastrointestinal Surgery, Renji Hospital Shanghai Jiao Tong University School of Medicine Shanghai China

**Keywords:** exhausted T cells, liver metastasis, primary colorectal cancer, regulatory T cells, single‐cell sequencing

## Abstract

Liver metastasis (LM) is an important factor leading to colorectal cancer (CRC) mortality. However, the effect of T‐cell exhaustion on LM in CRC is unclear. Single‐cell sequencing data derived from the Gene Expression Omnibus database. Data were normalized using the Seurat package and subsequently clustered and annotated into different cell clusters. The differentiation trajectories of epithelial cells and T cells were characterized based on pseudo‐time analysis. Single‐sample gene set enrichment analysis (ssGSEA) was used to calculate enrichment scores for different cell clusters and to identify enriched biological pathways. Finally, cell communication analysis was performed. Nine cell subpopulations were identified from CRC samples with LM. The proportion of T cells increased in LM. T cells can be subdivided into NK/T cells, regulatory T cells (Treg) and exhausted T cells (Tex). In LM, cell adhesion and proliferation activity of Tex were promoted. Epithelial cells can be categorized into six subpopulations. The transformation of primary CRC into LM involved two evolutionary branches of Tex cells. Epithelial cells two were at the beginning of the trajectory in CRC but at the end of the trajectory in CRC with LM. The receptor ligands CEACAM5 and ADGRE5‐CD55 played critical roles in the interactions between Tex and Treg cell‐epithelial cell, which may promote the epithelial‐mesenchymal transition process in CRC. Tex cells are able to promote the process of LM in CRC, which in turn promotes tumour development. This provides a new perspective on the treatment and diagnosis of CRC.

## 1 INTRODUCTION

Colorectal cancer (CRC) is the third most frequent malignant tumour of the digestive system with the second highest mortality rate. Recent decades have experienced an increase in the incidence of CRC.^1^, ^2^, ^3^ At present, immunotherapeutic strategies represented by immune checkpoint inhibitors could provide therapeutic options for patients with advanced CRC, though the overall efficacy is not satisfactory.^4^, ^5^, ^6^ Metastasis is a process during which cancer cells at the primary lesion spread to surrounding tissues and distant organs and distant metastasis is a major cause of cancer death. Liver metastasis (LM) is also a main cause of high mortality in CRC.^7^, ^8^ CRC patients with LM originating from the right and left sides of the colon manifests a survival discrepancy, despite the fact that left‐sided colon cancer normally has a higher prevalence of liver metastases.^7^ For CRC liver metastases, if detected in time, liver resection is the most effective treatment strategy, with a cure rate of up to 20% and more than 50% of the patients could survive for at least 5 years.^9^ CRC liver metastases have a relatively specific biology, which means that it is crucial to explore the biological mechanisms involved.

Conventionally, it is believed that tumours originating in epithelial tissues are susceptible to distal metastasis via lymph node metastasis.^10^ In mouse models, lymph node metastasis appears to assist distal metastasis of tumour cells.^11^ Accumulating evidence indicate that the interactions between epithelial cells and T cells play a critical role during LM in CRC. During tumour progression, tumour‐associated macrophages (TAMs) promote epithelial‐mesenchymal transition (EMT) via the TGF‐α signalling pathway, driving tumour cells to acquire mesenchymal properties and migrate to new metastatic sites.^12^ TGF‐α promotes angiogenesis, tumour growth and metastasis in CRC, especially in LM.^13^ These findings suggest that epithelial cells can regulate cancer cell LM through EMT transformation as well as the interaction with TAMs during the development of LM in CRC. In addition, tumour tissues also have abundant activated CD4^+^ T lymphocytes and CD8^+^ T lymphocytes during LM in CRC. In the presence of activated T cells, the killing effect of tumour cells becomes ‘selective’ and tumour cells could escape the killing action produced by T cells.^14^ Applying advanced next‐generation sequencing techniques, single‐cell RNA sequencing (scRNA‐Seq) enables the exploration of gene expression patterns in individual cells, unveiling the diversity within cell populations.^15^ ScRNA‐seq has been used to characterize CRC heterogeneity to develop a risk model for cancer based on mitochondrial autophagy‐mediated profiling.^16^ Also, Xu et al. used scRNA‐seq and observed a marked decrease in activated B cells in CRC with LM.^17^ Therefore, revealing the mechanism underlying LM occurrence based on scRNA‐seq technology might provide potential immunotherapeutic targets to facilitate the risk prediction for CRC patients.

The current research analysed scRNA‐seq data from primary CRC samples and CRC samples with LM to identify specific cell types and receptor/ligand factors involved in the interaction during LM in CRC. In addition, pseudo‐time analysis was conducted to characterize the differentiation trajectory of epithelial cells and potential mechanisms of epithelial cells during LM. In conclusion, our study may provide a new direction for the treatment of CRC.

## 2 MATERIALS AND METHODS

### 2.1 Raw data

The GSE231559 scRNA‐seq dataset was downloaded from the Gene Expression Omnibus website (https://www.ncbi.nlm.nih.gov/geo/). After removing mitochondrial, ribosomal and haemoglobin genes, the downstream data were read using the Read10X function of the Seurat package,^18^ and the SCTransform function was used for data normalization. Batch effect was removed by the harmony package^19^ (max.iter.harmony = 20, λ = 0.5). Next, based on the first 20 principal components, Uniform Manifold Approximation and Projection (UMAP) was performed for dimensionality reduction, and finally the cells were clustered into groups using the FindNeighbors and FindClusters functions at resolution = 0.1. Cell subpopulations were annotated using the marker genes provided by the CellMarker database (http://bio‐bigdata.hrbmu.edu.cn/CellMarker or http://117.50.127.228/CellMarker/).^20^

### 2.2 Pseudo‐time analysis

To characterize the mechanism of LM development in CRC, pseudo‐time trajectory analysis was conducted using Monocle.^21^ Differentially expressed gene set (|log2FC| >0.25, min.pct = 0.25) between different groups was compared using the FindMarkers function to construct differentiation trajectories. We used the newCellDataSet to create the cds object and filter low‐quality cells, followed by using reduceDimension function to reduce data size by running the DDRTree algorithm. The cells were then ordered by orderCells function and the trajectory plot was produced by the plot_cell_trajectory function. The plot_genes_in_pseudotime function showed a scatter plot of the correlation between the expression of genes of interest and pseudotime. The branch involving more normal cells was taken as the beginning and end of the trajectory.

### 2.3 Single‐sample enrichment analysis

The Hallmark dataset was downloaded from The Molecular Signatures Database (MSigDB, https://www.gsea‐msigdb.org/gsea/msigdb). Based on the function of GSVA package,^22^ the pathways related to cancer progression were extracted and single‐sample enrichment analysis (ssGSEA) was performed.

### 2.4 Cell communication analysis

To explore the types of interactions between cells in primary CRC and CRC with LM, cell communication analysis was conducted using the CellChat package.^23^ The probabilities of different cell types to produce signalling and cell–cell contact were inferred based on the CellChatDB database and overexpressed ligands or receptors in these cell types were identified.

### 2.5 Statistical analysis

We used the Wilcox test to compare the differences in continuous variables between the two groups. All calculations were performed in the R language (version 4.3.1). A *p* < 0.05 was considered statistically significant.

## 3 RESULTS

### 3.1 Cell types in primary CRC samples and CRC samples with LM

**FIGURE 1:**
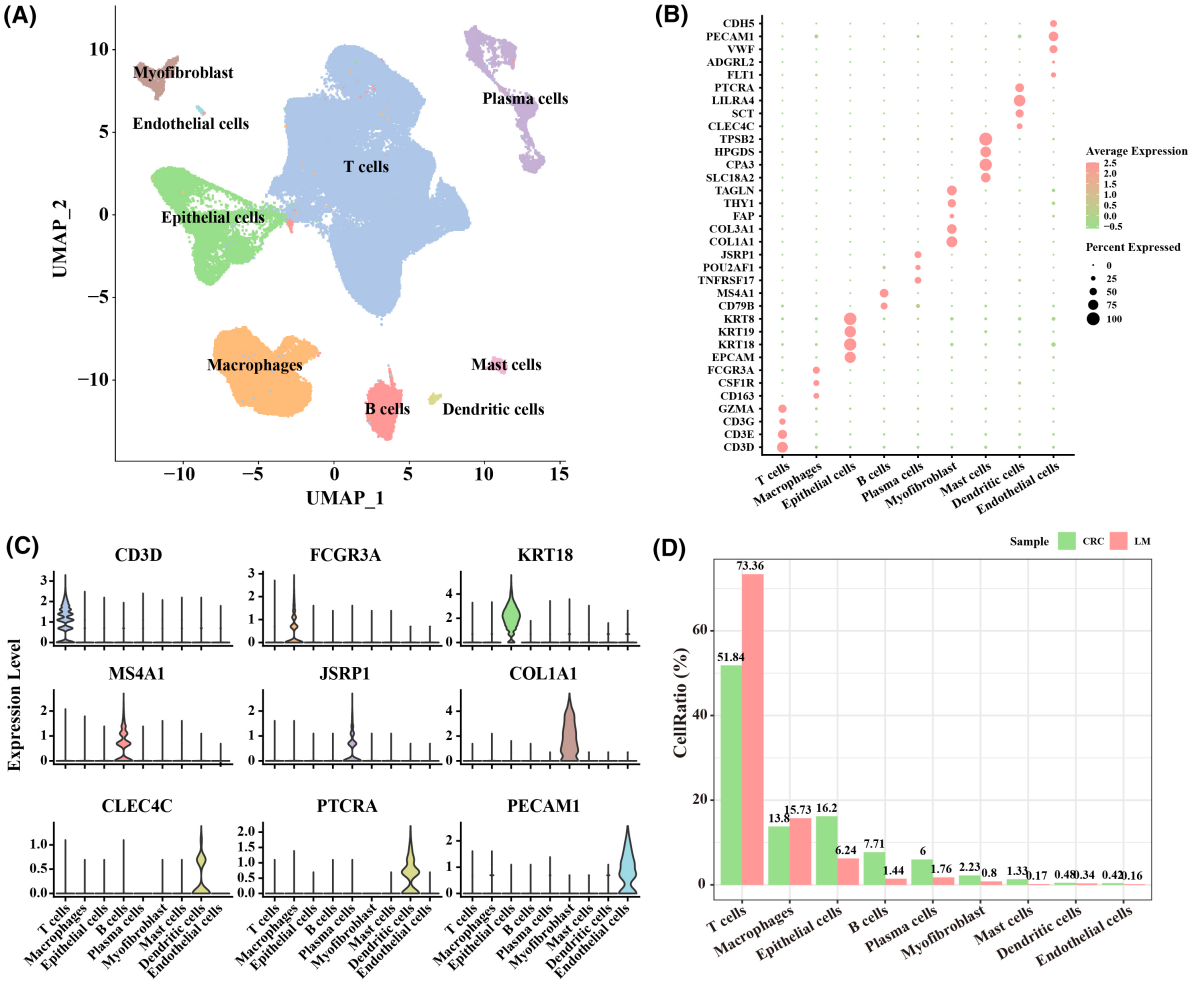
Cell types in primary colorectal cancer (CRC) and CRC liver metastatic tumour samples. (A) Nine cell subpopulations in primary CRC and CRC liver metastatic tumour samples. (B) Bubble diagram showing the highly expressed marker genes in the nine cell subpopulations. (C) Violin plot demonstrating highly expressed marker genes in nine cell subpopulations. (D) The proportion of nine cell subpopulations in primary CRC and liver metastasis (LM) samples.

To investigate cell populations in primary CRC and CRC samples with LM and relevant molecular features, these two types of samples were included in our scRNA‐seq data analysis. After clustering analysis, UMAP dimensionality reduction analysis and marker gene annotation were performed using the Seurat package. A total of 112,586 cells were grouped into nine cell subpopulations, namely, T cells (marked with GZMA, CD3G, CD3E and CD3D), macrophages (marked with FCGR3A, CSF1R and CD163), epithelial cells (marked with KRT8, KRT19, KRT18 and EPCAM), B cells (marked with MS4A1 and CD79B), plasma cells (marked with JSRP1, POU2AF1 and TNFRSF17), myofibroblast (marked with TAGLN, THY1, FAP, COL3A1 and COL1A1), mast cells (marked with TPSB2, HPGDS, CPA3 and SLC18A2), dendritic cells (marked with PTCRA, LILRA4, SCT and CLEC4C), endothelial cells (marked with CDH5, PECAM1, VWF, ADGRL2 and FLT1) (Figure 1A–C). The proportions of T cells and epithelial cells varied considerably in the two types of CRC samples. The proportion of T cells increased in CRC with LM (CRC: 51.8%, LM: 73.36) and that of epithelial cells decreased in CRC with LM (CRC: 16.2%, LM: 6.24%) (Figure 1D), indicating that T cells and epithelial cells played a crucial role in the process of LM in CRC.

### 3.2 T‐cell subpopulations in primary CRC and LM tumour samples

**FIGURE 2:**
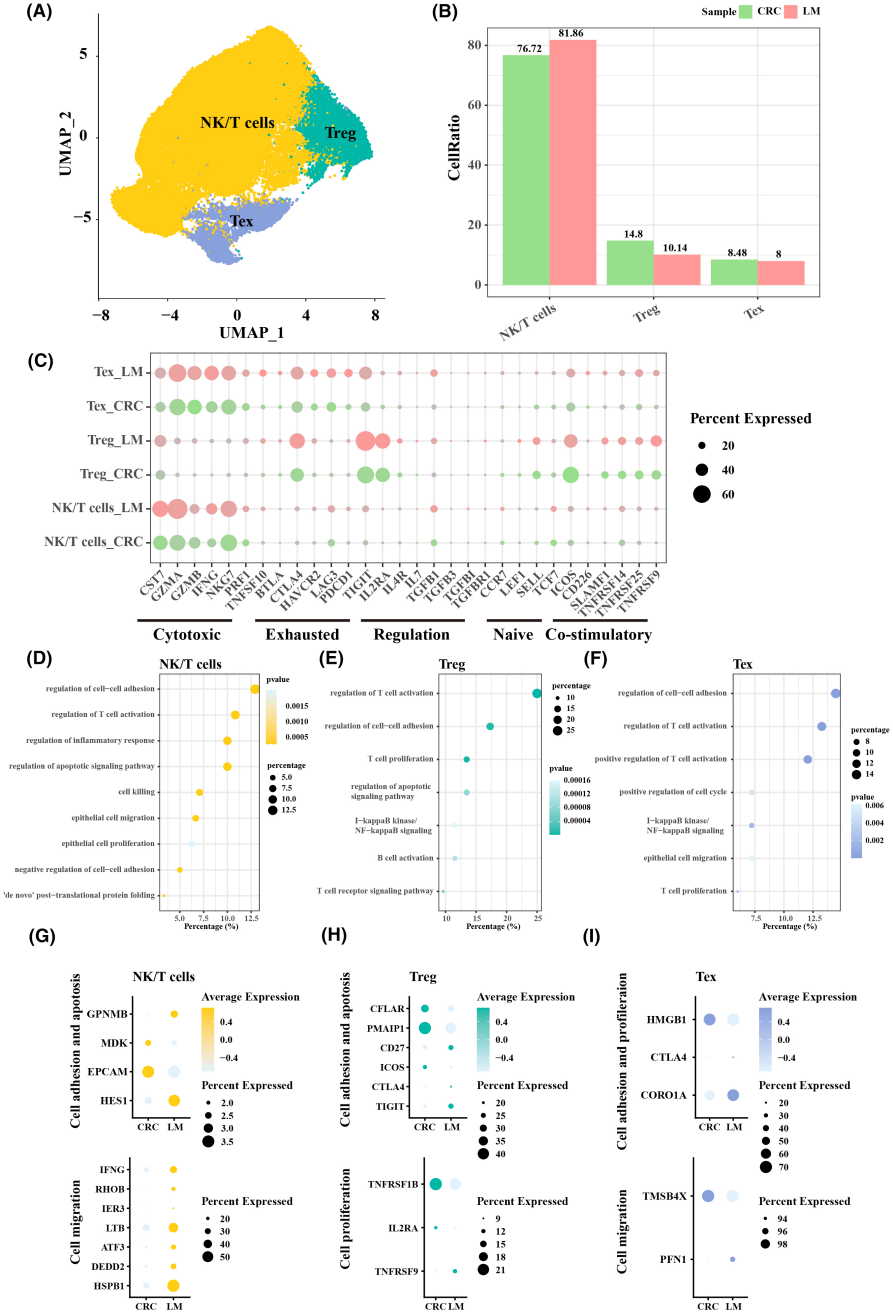
Landscape of T‐cells subpopulations in primary colorectal cancer (CRC) and liver metastasis (LM) tumour samples. (A) Three subpopulations in T cells, NK/T cells, Treg, and Tex. (B) The proportion of NK/T cells, Treg, and Tex in primary CRC and LM tumour samples. (C) The expression levels of cytotoxic factor, exhausted factor, regulation factor, naïve factor, and co‐stimulatory factor. (D) GO biological process involved in NK/T cells. (E) GO biological process involved in Treg. (F) GO biological process involved in Tex. (G) The expression levels of cell adhesion and apotosis‐related genes and cell proliferation‐related genes in NK/T cells. (H) The expression levels of cell adhesion and apotosis‐related genes and cproliferation‐related genes in Treg. (I) The expression levels of cell adhesion and profileraion‐related genes and cell migration‐related genes in Tex.

Subpopulation T cells was categorized according to the difference in the proportion of T cells in primary CRC and LM samples. Based on the expression annotation of marker genes, T cells were subdivided into natural killer (NK)/T cells, regulatory T cells (Treg) and exhausted T cells (Tex) (Figure 2A). Specifically, we found that the proportion of NK/T cells was higher in LM samples, while that of Tregs and Tex was higher in primary CRC samples (Figure 2B). Cytotoxic factors, including CST7, GZMA, GZMB, IFNG and NKG7, were upregulated in Tex_LM, Tex_CRC, NK/T cells_LM and NK/T cells_CRC (Figure 2C). GO biological process analysis was performed based on the differentially expressed genes (DEGs). Specifically, NK/T cells were involved in the regulation of cell–cell adhesion, regulation of T‐cell activation, regulation of inflammatory response and regulation of the apoptotic signalling pathway (Figure 2D); Treg was involved in regulation of T‐cell activation, regulation of cell–cell adhesion and T‐cell proliferation (Figure 2E); Tex was involved in regulation of cell–cell adhesion, regulation of T‐cell activation and positive regulation of T‐cell activation (Figure 2F). The expression levels of cell adhesion and apoptosis‐related genes, cell proliferation‐related genes and cell migration‐related genes were also evaluated in T‐cell subpopulations in primary CRC and LM tumour samples. It was found that the expression of cell adhesion and apoptosis‐related genes, cell migration‐related genes was upregulated in NK/T cells in LM samples (Figure 2G). The expression of genes related to cell adhesion, apoptosis and proliferation was upregulated in Treg in primary CRC samples (Figure 2H), while the expression of genes related to cell adhesion, proliferation (CORO1A) and migration (PFN1) was upregulated in Tex in LM (Figure 2I). These results suggested a possible involvement of these genes in the development of LM in CRC patients.

### 3.3 Epithelial cell subpopulations in primary CRC and LM tumour samples

**FIGURE 3:**
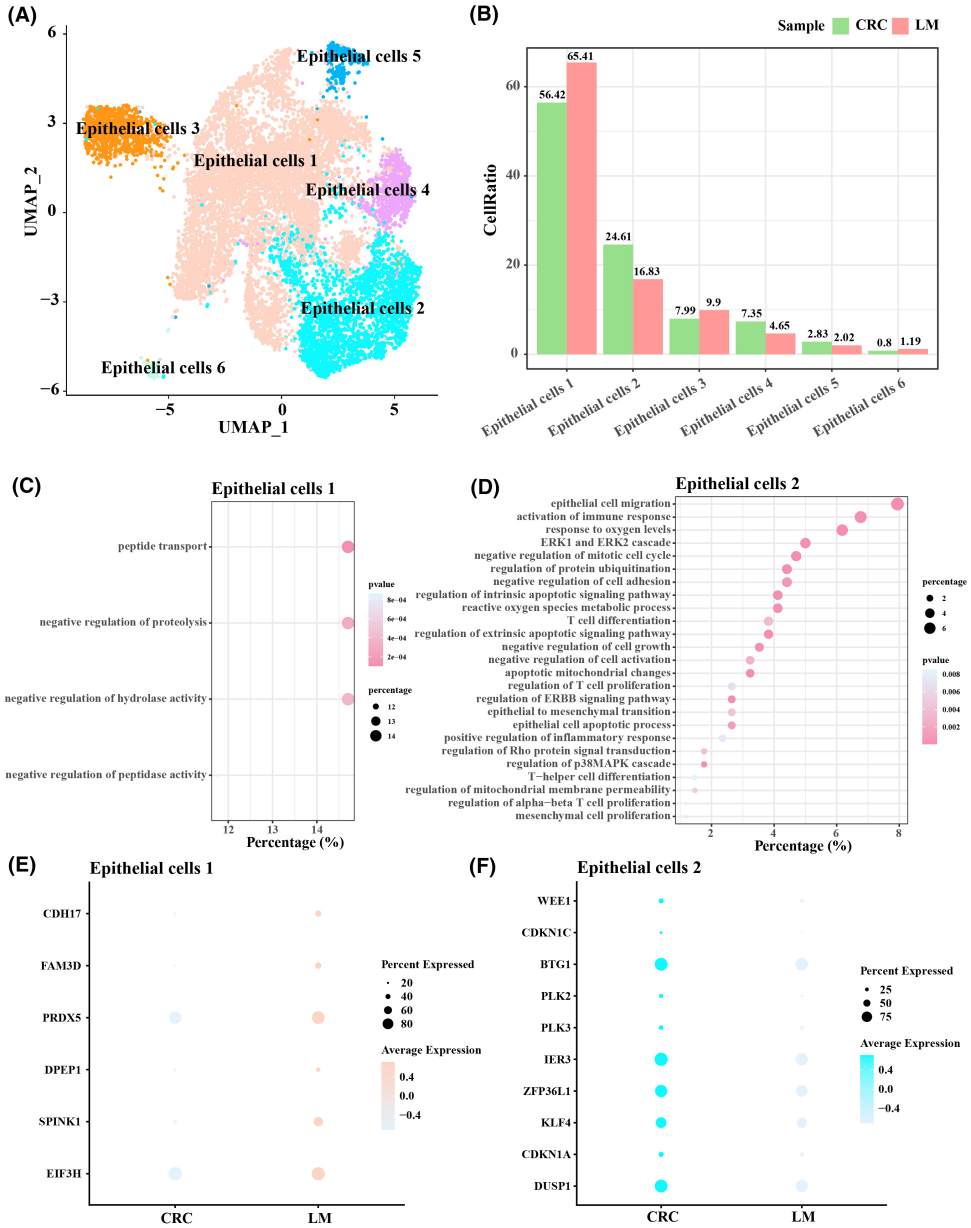
The subpopulations of epithelial cells in primary colorectal cancer (CRC) and liver metastasis (LM) tumour samples. (A) Six subpopulations in epithelial cells. (B) Proportion of the six subpopulations in primary CRC and LM tumour samples. (C) GO biological processes involved in epithelial cells 1. (D) GO biological process involved in epithelial cells 2. (E) The expression levels of protein metabolic activity‐related genes and transporter‐related genes. (F) The expression levels of epithelial cell igration‐related genes, epithelial mesenchymal transition‐related genes.

Epithelial cells could be categorized into six subpopulations, namely, epithelial cells 1, epithelial cells 2, epithelial cells 3, epithelial cells 4, epithelial cells 5 and epithelial cells 6 (Figure 3A). Epithelial cells 1 and 2 had higher proportions, with LM samples having more epithelial cells 1 and primary CRC samples having more epithelial cells 2 (Figure 3B). Analysis on the cellular functions showed that epithelial cells 1 were mainly associated with negative regulation of protein activity, transport, for example, peptide transport, negative regulation of proteolysis, negative regulation of hydrolase activity, negative regulation of peptidase activity (Figure 3C), while epithelial cells 2 were mainly associated with epithelial cell migration, epithelial mesenchymal transition, for example, mesenchymal cell proliferation, epithelial cell migration, epithelial to mesenchymal transition, negative regulation of cell adhesion (Figure 3D). These results were confirmed by gene expression patterns in epithelial cells 1 and epithelial cells 2 because protein metabolic activity‐related genes, and transporter‐related genes were expressed at higher levels in LM (Figure 3E), while epithelial cell migration‐related genes, and epithelial mesenchymal transition‐related genes were expressed at higher levels in primary CRC (Figure 3F). To conclude, epithelial cells may play an important role in the process of EMT to promote metastasis in CRC.

### 3.4 Pseudo‐time analysis for Tex cells

**FIGURE 4:**
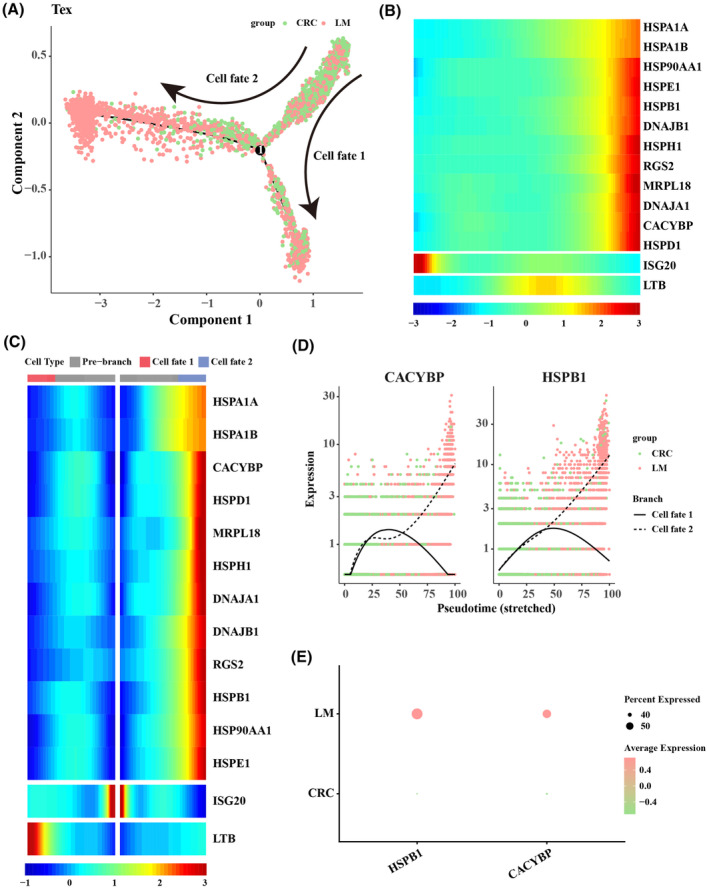
Pseudo‐time analysis for Tex cells. (A) Pseudo‐time differentiation trajectory of Tex cells. (B) Heatmap of the expression of tumour development‐related genes in the pseudo‐time trajectory of Tex cells. (C) Expression heatmap of tumour development‐related genes in cell fate 1 and cell fate 2 in the pseudo‐time trajectory of Tex cells. (D) Expression changes of CACYBP and HSPB1 in cell fate 1 and cell fate 2 with pseudo‐time progression. (E) Expression levels of CACYBP and HSPB1 in primary colorectal cancer (CRC) and liver metastasis (LM) samples.

Pseudo‐time analysis was performed to characterize the transformation of Tex cells and Tregs during LM, and the results revealed two evolutionary branches (cell fate 1 and cell fate 2) of Tex cells during the transformation of primary CRC to LM (Figure 4A). As cell fate 2 was more abundant in LM samples, therefore we further focused on the changes in the expression of tumour development‐related genes in cell fate 2. The results showed that the expression of CACYBP and HSPB1 in cell fate 2 gradually increased with pseudo‐time progression (Figure 4B–D). Correspondingly, the expression levels of CACYBP and HSPB1 were also higher in LM samples than those in primary CRC samples (Figure 4E).

### 3.5 Pseudo‐time analysis for epithelial cells 2

**FIGURE 5:**
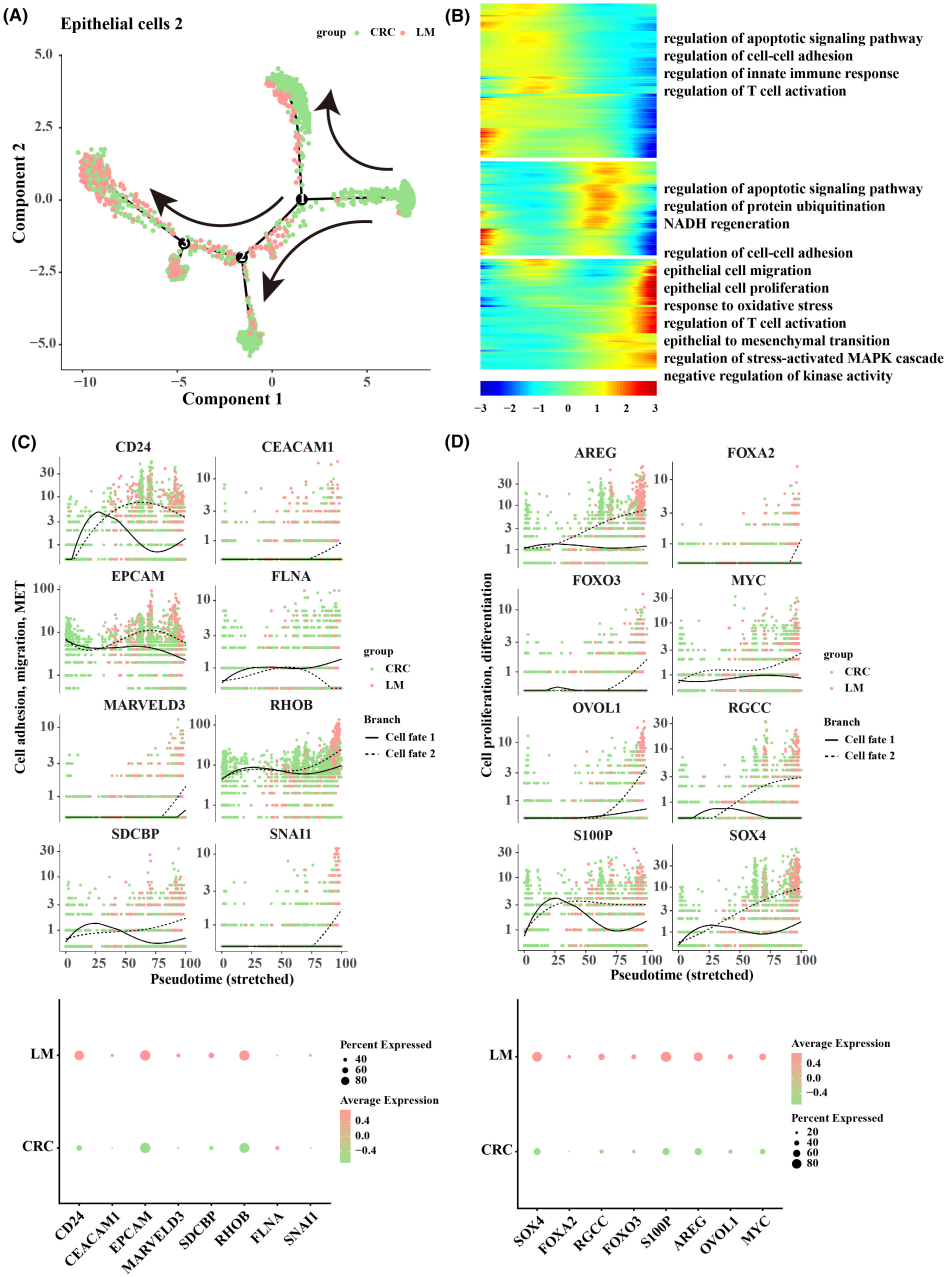
Pseudo‐time analysis for epithelial cells 2. (A) Pseudo‐time differentiation trajectory for epithelial cells 2. (B) Heatmap of tumour progression‐associated GO terms in the pseudo‐time trajectory for Epithelial cells 2. (C) Expression changes of genes related to cell adhesion, migration and mesenchymal transition with pseudo‐time progression in FATE 1 and FATE 2, different samples. (D) The expression changes of cell proliferation, differentiation‐related genes in the FATE 1 and FATE 2, different samples, with pseudo‐time progression.

During tumour metastasis to the liver in CRC, epithelial cells 2 were mainly associated with epithelial cell migration and epithelial mesenchymal transition. Therefore, to investigate the alteration of epithelial cells 2 during LM, pseudo‐time analysis was performed. The results showed that epithelial cells 2 in CRC were at the beginning of the trajectory and epithelial cells 2 in LM were at the end of the trajectory. During the differentiation process, there were two differentiation trajectories (FATE 1 and FATE 2), and eventually part of the epithelial cells 2 resulted in the phenomenon of LM in CRC (Figure 5A). The genes in the cells at the end of each branch were analysed by GO_biological process, and we found significantly enriched epithelial cell migration, epithelial cell proliferation, response to oxidative stress and regulation of T‐cell activation (Figure 5B). We also examined the differential expression of genes related to cell adhesion, migration, and mesenchymal transition in primary CRC and LM. The results demonstrated that the expression levels of CD24, CEACAM1, EPCAM, MARVELD3, RHOB, SDCBP and SNAI1 were higher in FATE 2, while CD24, CEACAM1 and MARVELD3 were expressed at higher levels in LM (Figure 5C). The expression trend of genes related to cell proliferation, and differentiation was also consistent, as we found that expression levels of AREG, FOXA2, FOXO3, MYC, OVOL1, RGCC, S100P and SOX4 were higher in FATE 2, while SOX4, S100P and AREG were expressed at higher levels in LM (Figure 5D).

### 3.6 Functional and cellular communication information on the involvement of epithelial cells 2 and Tex cell fate 2

**FIGURE 6:**
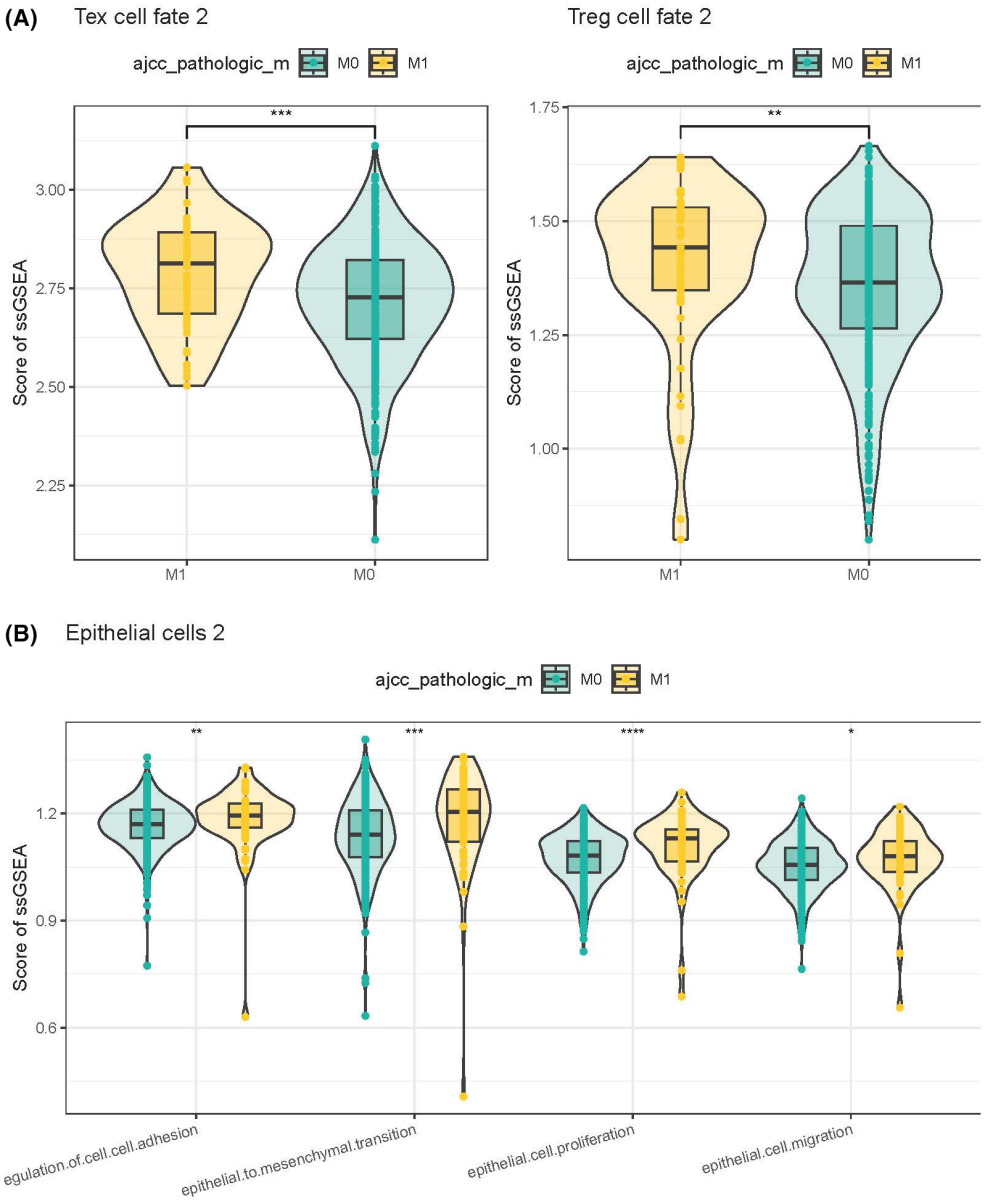
Functions involved in epithelial cells 2 and Tex cell fate 2. (A) Single‐sample gene set enrichment analysis (ssGSEA) enrichment scores for Tex cell fate 2 and Treg cell fate 2. (B) The ssGSEA enrichment score of Epithelial cells 2 in tumour metastasis‐related signalling pathways.

Analysis on the tumour metastasis‐related pathway activities of epithelial cells 2 and Tex cell fate 2 in patients with M0 and M1 stages showed that Tex cell fate 2 and Treg cell fate 2 might play important roles in metastatic tumours (Figure 6A). In epithelial cells 2, biological processes such as cell adhesion, mesenchymal transition, proliferation and migration played more important roles in metastatic tumours (Figure 6B).

**FIGURE 7:**
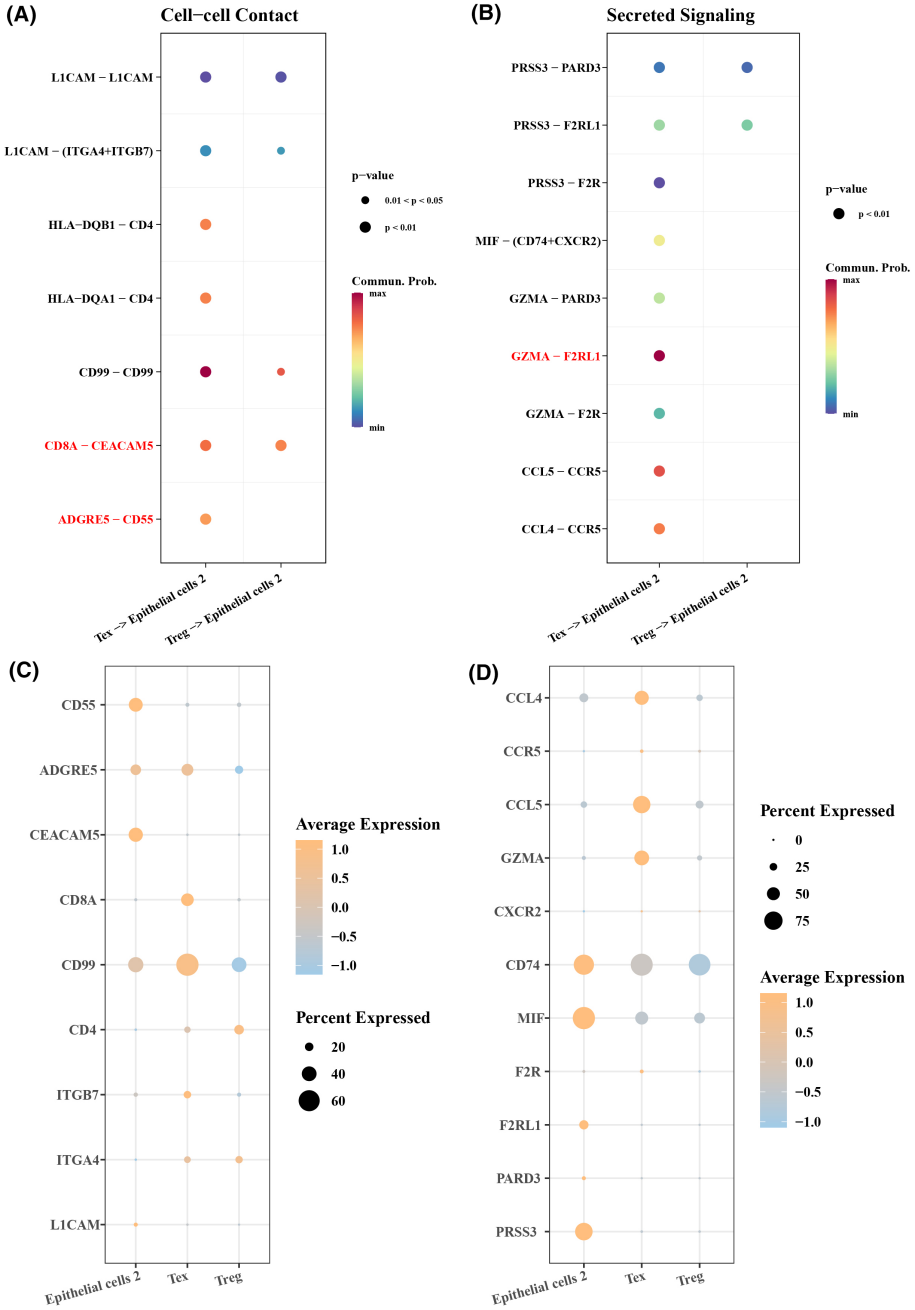
Epithelial cells 2 and Tex cell fate 2 cells exchange information. (A) Receptor‐ligand pairs in cell–cell contact, Tex and Treg in cell–cell contact with epithelial cells 2. (B) Secreted signalling, receptor‐ligand pairs for cell–cell contact of Tex and Treg with epithelial cells 2. (C) The expression of receptor‐ligand pairs for cell–cell contact of Tex and Treg with epithelial cells 2 in single‐cell data. (D) The expression of receptor‐ligand pairs of Tex and Treg for secreted signalling with epithelial cells 2 in single‐cell data.

Cell communication was analysed to further explore the interaction between Tex and Treg cells and epithelial cells 2 in promoting EMT. The results showed that in cell–cell contact, CD8A‐CEACAM5 and ADGRE5‐CD55 receptor ligand pairs had a greater possibility of interaction between Tex cells and epithelial cells 2 (Figure 7A), and CD8A, CEACAM5, ADGRE5 and CD55 exhibited a greater possibility of interaction between epithelial cells 2 and Tex cells (Figure 7C). In secreted signalling, the GZMA‐F2RL1 receptor‐ligand pair showed a greater possibility of interaction in Tex‐epithelial cells 2 (Figure 7B) and was high‐expressed inepithelial cells 2 and Tex (Figure 7D). These results suggested that Tex and Treg cells played an important role in epithelial cells during CRC development, indicating that Tex cells may be involved in tumour progression by altering the immune microenvironment in CRC.

## 4 DISCUSSION

In this study, we analysed scRNA‐seq data from primary CRC samples and CRC samples with LM. By constructing a single‐cell atlas, it was found that the proportion of T cells in the LM increased and that of epithelial cells decreased. Study shows that that liver sinusoidal endothelial cells capture cancer cells, while macrophages phagocytose cancer cells and release tumour‐killing cytokines.^24^ During this process, antigen‐presenting cells present antigens to T cells for conversion into effector T cells, and this process could be enhanced by CD4^+^ T cells. However, when cancer cells evade the immune system, effector T cells under the effect of immune checkpoints would become dysfunctional.^24^ In cases of preoperative chemotherapy for primary CRC, the abundance of T cells reduces significantly.^25^ EMT is a process during which epithelial cells lose the characteristic features and turn to malignant tumour cells with metastatic tendency, which could explain the decrease in the number of epithelial cells.^26^ Interactions between cells in the tumour microenvironment (TME) may increase the proportion of T cells and reduce the proportion of epithelial cells. The reduced proportion of these two types of cells may be responsible for the development of LM in CRC.

Studies have increasingly confirm that Tex cell formation undergoes metabolic deficiencies and is accompanied by overall alterations in signalling molecular pathways and epigenetics that could result in the suppression of effective immune responses and ineffectiveness of immune checkpoint therapies, partly contributing to tumour progression.^27^, ^28^ There were three molecular subtypes of T cells (Treg, Tex and NK cells), with Treg and Tex as the predominate subtypes. In the tumour mesenchyme, chemokines produced by malignant cells and some other cells attract Tregs from the circulation to reach the tumour site. Pre‐existing Tregs can be clonally amplified in response to specific antigen activation in the presence of TGF‐β and IL‐10, two abundant cytokines in the TME. These cytokines together with suboptimal antigen presentation promote the conversion of conventional T cells into Tregs with suppressive functions.^29^ Environmental factors also play an important role, and CRC often exhibits persistent antigenic stimulation and chronic inflammation in the TME. Chronic antigen exposure could increase excessive and sustain T‐cell activation, eventually leading to T‐cell depletion and the formation of Tregs.^30^ CFLAR and TNFRSF1B in Treg were high‐expressed in primary CRC. Treg cells have a higher apoptosis rate compared to conventional T cells, which is correlated with low c‐FLIP expression. Specific knockdown of c‐FLIP in Treg cells results in a fatal autoimmune disease that manifests as peripheral Treg cell deletion, effector cell activation, multi‐organ immune cell infiltration and premature death.^31^ TNFR2^+^ Treg cells preferentially accumulate in the TME and express high levels of immunosuppressive molecules. In an in vitro model, the TNF‐α/TNFR2 pathway upregulates Foxp3 expression in CD4^+^ CD25^+^ T cells and latent TGF‐β production in Treg and enhances the immunosuppressive function of Treg.^32^ These studies suggest that high expression of CFLAR and TNFRSF1B in Treg cells plays an important role in the regulation of the TME and progression of CRC, especially in regulating immune responses and promoting immune escape.

There were six cell subpopulations in epithelial cells, and epithelial cells 1 and epithelial cells 2 were the dominant cell types. Epithelial cells 1 was largely involved in the negative regulation of protein activity, and transport, while epithelial cells 2 were more associated with epithelial cell migration, epithelial mesenchymal transition. In cancers, the regulation of protein activity and transport in epithelial cells is closely related to cancer progression. PEPT1 and PEPT2 are important transporter proteins for the uptake of peptides and amino acids in intestinal renal epithelial cells, which regulate the uptake and metabolism of oxidative drugs and influence the therapeutic outcome of cancer.^33^, ^34^ Epithelial cells may undergo significant structural changes in the complex cytokine and chemokine environment within the TME, which may lead to loss of cellular function.^35^, ^36^ Epithelial cells 2 were mainly associated with epithelial cell migration, epithelial mesenchymal transition and were proportionally higher in primary CRC and had higher expression levels of BTG1, IER3, ZFP36L1 and DUSP1. Upregulated BTG1 expression can promote CRC cell proliferation.^37^ Blocking the IER3/Nrf2 pathway significantly enhances the effect of antitumor drugs on bladder cancer.^38^ Significantly overexpressed ZFP36L1 in highly malignant tumour cells causes a high accumulation of proliferative and migratory properties in tumour cells.^39^ DUSP1 is significantly overexpressed in apatinib‐resistant patients with gastric cancer, and downregulating DUSP1 may be a potential measure to attenuate apatinib resistance in gastric cancer.^40^ It should be noted that further studies are needed to elucidate the specific mechanisms underlying the high expression of epithelial cells 1 and epithelial cells 2 in CRC liver metastases.

This study revealed multiple paired transcripts that mediated the communication between Tex and Treg cells and epithelial cells, such as ADGRE5‐CD55 and CD8A‐CEACAM5. CEACAM5 as a molecule closely related to cell adhesion^41^ plays a key role in the therapeutic diagnosis and prognosis in a variety of epithelial tumours, including respiratory and gastrointestinal tumours.^42^ Gebauer et al. indicate that CEACAM5 overexpression can promote tumour growth through the EMT pathway in the localized regions of aggressive cancer.^43^ ADGRE5 (CD97) has been considered as the most promising target of adhesion G protein‐coupled receptors in glioblastoma.^44^ ADGRE5 in retinal pigment epithelial cells also controls leukocyte activation and trafficking in uveal retinal inflammation.^45^ CD55, a complement regulatory protein, binds to multiple ligands that are constitutively expressed on monocytes or granulocytes and is rapidly upregulated during activation of T cells.^46^ It has been shown that CD55 regulates both innate and adaptive immune responses and mediates T‐cell co‐stimulation when binding to CD97.^47^ This revealed a potential interaction between Tex and Treg cells in the colon epithelium, which in turn promoted mesenchymal transformation and the development of cancer cells. However, there were some limitations in our study. First, the main source of data came from public databases, suggesting that a broader amount of data have not been taken into analysis. In addition, further in vivo experiments are required to verify the reliability and accuracy of the current results.

## 5 CONCLUSION

Overall, our study analysed the heterogeneity of cells in CRC samples and CRC samples with LM, revealing essential cell types in the process of LM development. Tex cells promoted the process of LM in CRC and participated in tumour progression mainly by altering the immune microenvironment surrounding the tumour. The present findings expanded the understanding of LM in CRC, providing the mechanisms through which Tex and Treg cells interacted with epithelial cells. Single‐cell data identified diagnostically important and potential mechanistic features for CRC immunotherapy.

## AUTHOR CONTRIBUTIONS

**Tianlong Ling:** Conceptualization (lead); data curation (equal); formal analysis (equal); methodology (equal); resources (equal); software (equal); supervision (equal); writing – original draft (lead); writing – review and editing (lead). **Cheng Zhang:** Investigation (equal); methodology (equal); project administration (equal); resources (equal); software (equal); writing – original draft (lead); writing – review and editing (lead). **Ye Liu:** Conceptualization (equal); investigation (equal); methodology (equal); validation (equal). **Chunhui Jiang:** Investigation (equal); methodology (equal); software (equal); supervision (equal); validation (equal); visualization (equal). **Lei Gu:** Conceptualization (equal); data curation (equal); formal analysis (equal); investigation (equal); resources (equal); software (equal); supervision (equal); validation (equal).

## FUNDING INFORMATION

The authors declare that they received no funding.

## CONFLICT OF INTEREST STATEMENT

The authors declare that they have no conflicts of interest regarding this manuscript.

## Data Availability

The datasets generated and/or analysed during the current study are available in the [GSE231559] repository, [https://www.ncbi.nlm.nih.gov/geo/query/acc.cgi?acc= GSE231559].

## References

[1] Sung H , Ferlay J , Siegel RL , et al. Global cancer statistics 2020: GLOBOCAN estimates of incidence and mortality worldwide for 36 cancers in 185 countries. CA Cancer J Clin. 2021;71(3):209‐249. 33538338 10.3322/caac.21660

[2] Xia C , Dong X , Li H , et al. Cancer statistics in China and United States, 2022: profiles, trends, and determinants. Chin Med J. 2022;135(5):584‐590. 35143424 10.1097/CM9.0000000000002108 PMC8920425

[3] Ma S , Zhu X , Xin C , et al. RCN3 expression indicates prognosis in colorectal cancers. Oncologie. 2022;24(4):823‐833.

[4] Weng J , Li S , Zhu Z , et al. Exploring immunotherapy in colorectal cancer. J Hematol Oncol. 2022;15(1):95. 35842707 10.1186/s13045-022-01294-4 PMC9288068

[5] Kalbasi A , Ribas A . Tumour‐intrinsic resistance to immune checkpoint blockade. Nat Rev Immunol. 2020;20(1):25‐39. 31570880 10.1038/s41577-019-0218-4 PMC8499690

[6] de Miguel M , Calvo E . Clinical challenges of immune checkpoint inhibitors. Cancer Cell. 2020;38(3):326‐333. 32750319 10.1016/j.ccell.2020.07.004

[7] Engstrand J , Nilsson H , Stromberg C , Jonas E , Freedman J . Colorectal cancer liver metastases–a population‐based study on incidence, management and survival. BMC Cancer. 2018;18(1):78. 29334918 10.1186/s12885-017-3925-x PMC5769309

[8] Huang S , Zhang R , Liu L . Comprehensive network analysis of the molecular regulation mechanism for breast cancer metastasis. Oncologie. 2021;23(1):159‐171.

[9] Padmanabhan C , Nussbaum DP , D'Angelica M . Surgical Management of Colorectal Cancer Liver Metastases. Surg Oncol Clin N Am. 2021;30(1):1‐25. 33220799 10.1016/j.soc.2020.09.002

[10] Jin K , Li T , van Dam H , Zhou F , Zhang L . Molecular insights into tumour metastasis: tracing the dominant events. J Pathol. 2017;241(5):567‐577. 28035672 10.1002/path.4871

[11] Pereira ER , Kedrin D , Seano G , et al. Lymph node metastases can invade local blood vessels, exit the node, and colonize distant organs in mice. Science. 2018;359(6382):1403‐1407. 29567713 10.1126/science.aal3622 PMC6002772

[12] Gazzillo A , Polidoro MA , Soldani C , Franceschini B , Lleo A , Donadon M . Relationship between epithelial‐to‐mesenchymal transition and tumor‐associated macrophages in colorectal liver metastases. Int J Mol Sci. 2022;23(24):16197. 36555840 10.3390/ijms232416197 PMC9783529

[13] Chruscik A , Gopalan V , Lam AK . The clinical and biological roles of transforming growth factor beta in colon cancer stem cells: a systematic review. Eur J Cell Biol. 2018;97(1):15‐22. 29128131 10.1016/j.ejcb.2017.11.001

[14] Wagner P , Koch M , Nummer D , et al. Detection and functional analysis of tumor infiltrating T‐lymphocytes (TIL) in liver metastases from colorectal cancer. Ann Surg Oncol. 2008;15(8):2310‐2317. 18521684 10.1245/s10434-008-9971-5

[15] Potter SS . Single‐cell RNA sequencing for the study of development, physiology and disease. Nat Rev Nephrol. 2018;14(8):479‐492. 29789704 10.1038/s41581-018-0021-7 PMC6070143

[16] Gao H , Zou Q , Ma L , et al. Unveiling mitophagy‐mediated molecular heterogeneity and development of a risk signature model for colorectal cancer by integrated scRNA‐seq and bulk RNA‐seq analysis. Gastroenterol Rep. 2023;11:goad066. 10.1093/gastro/goad066 PMC10598840 37886241

[17] Xu Y , Wei Z , Feng M , et al. Tumor‐infiltrated activated B cells suppress liver metastasis of colorectal cancers. Cell Rep. 2022;40(9):111295. 36044847 10.1016/j.celrep.2022.111295

[18] Stuart T , Butler A , Hoffman P , et al. Comprehensive integration of single‐cell data. Cell. 2019;177(7):1888‐1902 e21. 31178118 10.1016/j.cell.2019.05.031 PMC6687398

[19] Korsunsky I , Millard N , Fan J , et al. Fast, sensitive and accurate integration of single‐cell data with harmony. Nat Methods. 2019;16(12):1289‐1296. 31740819 10.1038/s41592-019-0619-0 PMC6884693

[20] Hu C , Li T , Xu Y , et al. CellMarker 2.0: an updated database of manually curated cell markers in human/mouse and web tools based on scRNA‐seq data. Nucleic Acids Res. 2023;51(D1):D870‐D876. 36300619 10.1093/nar/gkac947 PMC9825416

[21] Trapnell C , Cacchiarelli D , Qiu X . Monocle: cell counting, differential expression, and trajectory analysis for single‐cell RNA‐Seq experiments. 2018.

[22] Hanzelmann S , Castelo R , Guinney J . GSVA: gene set variation analysis for microarray and RNA‐seq data. BMC Bioinformatics. 2013;14:7. 23323831 10.1186/1471-2105-14-7 PMC3618321

[23] Jin S , Guerrero‐Juarez CF , Zhang L , et al. Inference and analysis of cell‐cell communication using CellChat. Nat Commun. 2021;12(1):1088. 33597522 10.1038/s41467-021-21246-9 PMC7889871

[24] Wang Y , Zhong X , He X , et al. Liver metastasis from colorectal cancer: pathogenetic development, immune landscape of the tumour microenvironment and therapeutic approaches. J Exp Clin Cancer Res. 2023;42(1):177. 37480104 10.1186/s13046-023-02729-7 PMC10362774

[25] Che LH , Liu JW , Huo JP , et al. A single‐cell atlas of liver metastases of colorectal cancer reveals reprogramming of the tumor microenvironment in response to preoperative chemotherapy. Cell Discov. 2021;7(1):80. 34489408 10.1038/s41421-021-00312-y PMC8421363

[26] Xu H , Lan Q , Huang Y , et al. The mechanisms of colorectal cancer cell mesenchymal‐epithelial transition induced by hepatocyte exosome‐derived miR‐203a‐3p. BMC Cancer. 2021;21(1):718. 34147083 10.1186/s12885-021-08419-x PMC8214778

[27] Xiao Y , Jiang J , Chen Y , Huang Y , Wei C . PD‐1 relevant genes predict the prognosis of breast cancer and their prediction effect in tumor response to immunotherapy. Oncologie. 2022;24(4):729‐742.

[28] Wherry EJ , Kurachi M . Molecular and cellular insights into T cell exhaustion. Nat Rev Immunol. 2015;15(8):486‐499. 26205583 10.1038/nri3862 PMC4889009

[29] Mougiakakos D . Regulatory T cells in colorectal cancer: from biology to prognostic relevance. Cancers (Basel). 2011;3(2):1708‐1731. 24212779 10.3390/cancers3021708 PMC3757386

[30] Wang JC , Xu Y , Huang ZM , Lu XJ . T cell exhaustion in cancer: mechanisms and clinical implications. J Cell Biochem. 2018;119(6):4279‐4286. 29274296 10.1002/jcb.26645

[31] Plaza‐Sirvent C , Schuster M , Neumann Y , et al. c‐FLIP expression in Foxp3‐expressing cells is essential for survival of regulatory T cells and prevention of autoimmunity. Cell Rep. 2017;18(1):12‐22. 28052242 10.1016/j.celrep.2016.12.022

[32] Qu Y , Wang X , Bai S , et al. The effects of TNF‐alpha/TNFR2 in regulatory T cells on the microenvironment and progression of gastric cancer. Int J Cancer. 2022;150(8):1373‐1391. 34766338 10.1002/ijc.33873 PMC9298834

[33] Schniers BK , Rajasekaran D , Korac K , Sniegowski T , Ganapathy V , Bhutia YD . PEPT1 is essential for the growth of pancreatic cancer cells: a viable drug target. Biochem J. 2021;478(20):3757‐3774. 34569600 10.1042/BCJ20210377 PMC8589330

[34] Warsi J , Hosseinzadeh Z , Elvira B , et al. USP18 sensitivity of peptide transporters PEPT1 and PEPT2. PLoS One. 2015;10(6):e0129365. 26046984 10.1371/journal.pone.0129365 PMC4457862

[35] Akrida I , Bravou V , Papadaki H . The deadly cross‐talk between hippo pathway and epithelial‐mesenchymal transition (EMT) in cancer. Mol Biol Rep. 2022;49(10):10065‐10076. 35604626 10.1007/s11033-022-07590-z

[36] Pedersen SF , Hoffmann EK , Novak I . Cell volume regulation in epithelial physiology and cancer. Front Physiol. 2013;4:233. 24009588 10.3389/fphys.2013.00233 PMC3757443

[37] Su C , Huang DP , Liu JW , Liu WY , Cao YO . miR‐27a‐3p regulates proliferation and apoptosis of colon cancer cells by potentially targeting BTG1. Oncol Lett. 2019;18(3):2825‐2834. 31452761 10.3892/ol.2019.10629 PMC6676402

[38] Mao MH , Huang HB , Zhang XL , Li K , Liu YL , Wang P . Additive antitumor effect of arsenic trioxide combined with intravesical bacillus Calmette‐Guerin immunotherapy against bladder cancer through blockade of the IER3/Nrf2 pathway. Biomed Pharmacother. 2018;107:1093‐1103. 30257321 10.1016/j.biopha.2018.08.057

[39] Ding K , Zhang F , Qi G , et al. ZFP36L1 promotes gastric cancer progression via regulating JNK and p38 MAPK signaling pathways. Recent Pat Anticancer Drug Discov. 2023;18(1):80‐91. 35611776 10.2174/1574892817666220524102403

[40] Teng F , Xu Z , Chen J , et al. DUSP1 induces apatinib resistance by activating the MAPK pathway in gastric cancer. Oncol Rep. 2018;40(3):1203‐1222. 29956792 10.3892/or.2018.6520 PMC6072387

[41] Blumenthal RD , Leon E , Hansen HJ , Goldenberg DM . Expression patterns of CEACAM5 and CEACAM6 in primary and metastatic cancers. BMC Cancer. 2007;7:2. 17201906 10.1186/1471-2407-7-2 PMC1769503

[42] Zhang X , Han X , Zuo P , Zhang X , Xu H . CEACAM5 stimulates the progression of non‐small‐cell lung cancer by promoting cell proliferation and migration. J Int Med Res. 2020;48(9):300060520959478. 32993395 10.1177/0300060520959478 PMC7536504

[43] Gebauer F , Wicklein D , Horst J , et al. Carcinoembryonic antigen‐related cell adhesion molecules (CEACAM) 1, 5 and 6 as biomarkers in pancreatic cancer. PLoS One. 2014;9(11):e113023. 25409014 10.1371/journal.pone.0113023 PMC4237406

[44] Ravn‐Boess N , Roy N , Hattori T , et al. The expression profile and tumorigenic mechanisms of CD97 (ADGRE5) in glioblastoma render it a targetable vulnerability. Cell Rep. 2023;42(11):113374. 37938973 10.1016/j.celrep.2023.113374 PMC10841603

[45] Eichler W , Lohrenz A , Simon KU , et al. The role of ADGRE5/CD97 in human retinal pigment epithelial cell growth and survival. Ann N Y Acad Sci. 2019;1456(1):64‐79. 31397926 10.1111/nyas.14210

[46] Capasso M , Durrant LG , Stacey M , Gordon S , Ramage J , Spendlove I . Costimulation via CD55 on human CD4+ T cells mediated by CD97. J Immunol. 2006;177(2):1070‐1077. 16818763 10.4049/jimmunol.177.2.1070

[47] Abbott RJ , Spendlove I , Roversi P , et al. Structural and functional characterization of a novel T cell receptor co‐regulatory protein complex, CD97‐CD55. J Biol Chem. 2007;282(30):22023‐22032. 17449467 10.1074/jbc.M702588200

